# Importance of computed tomography angiography in acute/hyperacute ischemic stroke

**DOI:** 10.1590/0100-3984.2020.0168

**Published:** 2021

**Authors:** Bruna Arrais Dias, Karenn Barros Bezerra, Alexandre Sérgio de Araújo Bezerra, Vanessa Garcia Santana, Raquel Rodrigues Borges, Juliana Cavalcanti de Freitas Reinaux, Daniel Lima Souza, Fernando Bisinoto Maluf

**Affiliations:** 1 Hospital Santa Marta (HSM), Brasília, DF, Brazil.; 2 Hospital Universitário de Brasília, Brasília, DF, Brazil.; 3 Universidade de Brasília (UnB), Brasília, DF, Brazil.

**Keywords:** Brain ischemia, Stroke/etiology, Brain infarction, Computed tomography angiography, Cerebral arteries/diagnostic imaging, Isquemia encefálica, Acidente vascular cerebral/etiologia, Infarto encefálico, Angiotomografia por tomografia computadorizada, Artérias cerebrais/diagnóstico por imagem

## Abstract

**Objective:**

To evaluate the importance of computed tomography and computed tomography angiography (CTA) in stroke protocols, as well as their impact on endovascular treatment and on the determination of the etiology.

**Materials and Methods:**

Were evaluated 28 patients with acute/hyperacute stroke in the anterior circulation who underwent intracranial and cervical CTA between April 2018 and August 2019. The parameters evaluated were the degree of stenosis, plaque characteristics, type of infarct, treatment, etiology, and the Alberta Stroke Program Early CT Score (ASPECTS).

**Results:**

Of the 28 patients evaluated, 16 (57.1%) had an ASPECTS of 10 (the maximum score, indicative of normality). Four patients (14.3%) underwent thrombolytic treatment, and seven (25.0%) underwent mechanical thrombectomy. The etiology was atherosclerosis in 32.1% of the patients, cerebral small-vessel disease in 7.1%, cardioembolic in 7.1%, and undetermined in 53.6%. Regarding plaque, 17.9% of the patients presented stenosis ≥ 50%, 21.4% presented stable plaques, and 42.9% presented vulnerable plaques. Patients with a lower ASPECTS were more likely to have relevant stenosis and were more likely to have a total infarct.

**Conclusion:**

In the evaluation of patients with acute/hyperacute strokes, CTA provides important information, identifying occlusion, as well as helping define the etiology and inform decisions regarding treatment.

## Abstract (pt)

**Objetivo:**

Avaliar a importância da tomografia computadorizada e angiotomografia computadorizada (ATC) no protocolo de acidente vascular encefálico (AVE) e o seu impacto no tratamento endovascular e na determinação da etiologia.

Materiais e Métodos: Foram avaliados 28 pacientes com AVE agudo/hiperagudo da circulação anterior que realizaram ATC intracraniana e cervical, no período de abril de 2018 a agosto de 2019. Os parâmetros avaliados foram grau de estenose, placa, tipo do infarto, tratamento, etiologia e classificação Alberta Stroke Program Early CT Score (ASPECTS).

**Resultados:**

A maioria dos casos (16; 57,1%) apresentou ASPECTS de 10. Quatro pacientes (14,3%) realizaram tratamento trombolítico e sete (25%) foram submetidos a trombectomia mecânica. A etiologia foi aterosclerose em 32,1% dos pacientes, doença de pequenas artérias em 7,1%, cardioembólico em 7,1% e indeterminada em 53,6%. Em relação à placa, 17,9% apresentaram estenose maior que 50%, 21,4% apresentaram placas estáveis e 42,9%, placas instáveis. Pacientes com ASPECTS mais baixo apresentavam maior probabilidade de ter estenose relevante e apresentavam maior chance de ocorrência de infarto total.

**Conclusão:**

A ATC fornece informações importantes na avaliação do paciente com AVE agudo/hiperagudo, identificando a oclusão e auxiliando na definição da etiologia e no direcionamento do tratamento.

## INTRODUCTION

Unenhanced computed tomography (CT) of the skull is the initial examination of choice in the evaluation of patients with stroke, used in order to exclude intracranial hemorrhage and other diseases that mimic cerebral ischemia, as well as to identify the early signs and determine the extent of ischemia^**(1,2)**^.

The extent of early ischemic changes, as evaluated in a standardized manner with the Alberta Stroke Program Early CT Score (ASPECTS) classification, correlates with the infarct size in follow-up examinations and predicts the risk of hemorrhagic transformation^**(3,4)**^. Widely used in clinical practice, this classification guides the definition of treatment and the evaluation of patient prognosis within the context of acute stroke, lower scores being associated with worse outcomes^**(4,5)**^. The ASPECTS classification was initially designed to identify patients who would benefit from intravenous thrombolysis and was then extended to select patients for mechanical thrombectomy^**(6,7)**^.

Initial studies showed that the ASPECTS cutoff to identify patients in whom thrombolytic treatment would improve the prognosis would be > 7^**(8)**^. However, recent studies have shown that patients with an ASPECTS between 5 and 7 can also benefit from the treatment^**(7,9,10)**^.

Given the wide availability, speed of image acquisition, and low cost of CT^**(1,5)**^, randomized multicenter studies have demonstrated that the addition of CT angiography (CTA) to the protocol for patients with acute stroke results in only a slight increase of time and cost in comparison with magnetic resonance imaging^**(5)**^, without significantly delaying the initiation of treatment^**(2)**^.

The use of CTA provides additional information that is important for the early diagnosis and treatment of patients with stroke, identifying the occlusion of large vessels, the location/extent of clotting^**(2)**^, extracranial stenosis, and ischemic changes in the brain parenchyma^**(5)**^. The CTA findings also facilitate the selection of patients for mechanical thrombectomy, directing endovascular intervention only to the occluded vessel, thus precluding the need for cerebral angiography of nontarget vessels^**(11)**^. Another well-established advantage of CTA is that it makes it possible to evaluate the vessel anatomy prior to planning endovascular procedures^**(12)**^.

Early CTA evaluation of intracranial and extracranial circulation helps identify the cause of cerebral ischemia, which enables early institution of reperfusion treatment and helps reduce morbidity, as well as facilitating the definition of treatment for secondary prevention, thus reducing the likelihood of stroke recurrence^**(13,14)**^.

The aim of this study was to evaluate the importance of including CT and CTA in the stroke protocol, examining their role in the early identification of hyperacute/acute ischemic brain injury. We also attempted to determine their impact on the early initiation of treatment with intravenous thrombolysis and mechanical thrombectomy, as well as their role in determining the cause of the ischemic event.

## MATERIALS AND METHODS

In this study, we included patients with hyperacute/acute ischemic stroke in the anterior circulation who were treated in the emergency department and underwent unenhanced intracranial/cervical CT and CTA within the first 24 hours after the onset of symptoms, between April 2018 and August 2019. The exclusion criteria were the presence of hemorrhage or a tumor; the impossibility of performing CTA due to iodinated contrast allergy or impaired renal function (creatinine clearance < 30 in patients not on dialysis); and transient ischemic attack (complete resolution of symptoms within 24 hours of onset and no changes on imaging examinations).

Unenhanced CT examinations and CTA examinations were performed in a Philips 64-slice CT scanner (Brilliance; Philips Medical Systems, Amsterdam, the Netherlands). The image acquisition parameters were a tube voltage of 120 kVp, a tube current of 300 mAs, and a slice thickness of 5 mm. After administration of 1.3-1.5 mL/kg of nonionic iodinated contrast media (Optiray 350; Gerbet, Paris, France) with an injection pump at a flow rate of 4.5 mL/s, followed by a bolus injection of 30 mL at a flow rate of 5.5 mL/s, CTA images were acquired from the aortic arch to the intracranial cortical vessels.

### Analysis

The extent of early ischemic changes was analyzed using the ASPECTS classification, which divides the area supplied by middle cerebral artery (MCA) into ten anatomic regions^**(4,15)**^, of which three are subcortical (the caudate nucleus, lentiform nucleus, and internal capsule) and seven are cortical (M1-M6 and the insula). The baseline ASPECTS is 10, one point being subtracted for each area with early ischemic changes, characterized by hypoattenuation with loss of cortical-subcortical differentiation or focal edema^**(16)**^.

The most severe atherosclerotic plaque ipsilateral to the ischemic lesion was evaluated in terms of the degree of stenosis and the type of plaque. The degree of stenosis was quantified by the North American Symptomatic Carotid Endarterectomy Trial method, as follows—< 50%; 50-70%; > 70%; or total occlusion—stenosis ≥ 50% being considered relevant, in accordance with the Trial of Org 10172 in Acute Stroke Treatment (TOAST) criterion^**(17)**^. Plaques were also classified, by the degree of attenuation, as calcified (> 130 HU), mixed (50-130 HU), or fatty-fibrous (< 50 HU). The plaque surface was classified as regular or irregular and as ulcerated or not. Plaques were thereby divided into two categories^**(18)**^: stable (calcified with a regular surface); and vulnerable (mixed or fatty-fibrous, with an irregular surface and with or without ulceration).

According to the TOAST classification system, we divided ischemic stroke into five subtypes, by etiology^**(17)**^: large-artery atherosclerosis; cardioembolism; small-vessel occlusion; stroke of other determined etiology; and stroke of undetermined etiology. We defined large-artery atherosclerosis as relevant atherosclerosis or occlusion of intracranial or extracranial arteries ipsilateral to the cerebral ischemia and affecting an area > 1.5 cm, assuming that cardiac causes had been excluded^**(17,19,20)**^.

The cause of stroke was classified as cardioembolic when the embolism was determined to have originated in the heart. That determination was based on the absence of relevant cervical atherosclerosis and the existence of risk factors such as having a mechanical prosthetic heart valve, non-paroxysmal atrial fibrillation, intracavitary thrombus, dilated cardiomyopathy, and endocarditis^**(17)**^. When the affected area was ≤ 1.5 cm and there was no relevant atherosclerosis in the cervical artery ipsilateral to the infarct or the source was potentially cardioembolic, the patient was classified as having cerebral small-vessel disease (lacunar infarct).

The “stroke of other determined etiology” category covers causes unlike those mentioned above, such as non-atherosclerotic vascular disease (moyamoya disease or arterial dissection), hematological disorder, coagulopathy, and vasculitis. We defined stroke as being of undetermined etiology when there were two likely causes or a specific cause was not identified^**(17,19,20)**^.

On the basis of the Oxfordshire Community Stroke Project classification of imaging aspects^**(21)**^, we categorized cerebral infarcts as follows: total anterior circulation infarct (TACI); partial anterior circulation infarct (PACI); lacunar infarct; infarct in the centrum semiovale; or border-zone infarct. Posterior circulation infarcts were not included in the present study.

The TACI category includes the following^**(21)**^: infarct occupying the entire region supplied by the internal carotid artery (ICA); infarct involving more than one third of the area supplied by the MCA; and cortical infarct in the areas supplied by the anterior cerebral artery (ACA) or MCA, accompanied by ipsilateral infarct of the basil ganglia in the area supplied by the MCA. The PACI category includes infarcts that do not meet the criteria for TACI or lacunar infarct and are located in the areas supplied by the MCA or ACA. The lacunar infarct category includes infarcts ≤ 1.5 cm that are located in the deep white matter, basal ganglia, or brainstem^**(21)**^. Border-zone infarcts are those that are located in a transition zone between areas supplied by two or three arteries^**(21)**^.

### Statistical analysis

We analyzed the data using the program R, version 3.1.2 (The R Foundation for Statistical Computing, Vienna, Austria). Categorical variables are expressed as absolute and relative frequencies, whereas continuous variables are expressed as descriptive statistics.

We evaluated parameters related to stenosis, plaque, the type of infarct, treatment, cause, age, and ASPECTS. For the purposes of our statistical analysis, because the patient sample was small (N = 28), we chose to dichotomize the type of infarct as “total” or “partial”, the ASPECTS as ≤ 7 or > 7, and the following parameters as “yes” or “no”: relevant stenosis; vulnerable plaque; specific treatment; atherosclerosis as cause; and age ≥ 65 years.

Fisher’s exact test was used in order to determine whether the dichotomized ASPECTS correlated with the parameters stenosis, plaque, infarct type, treatment, etiology, and age. The same parameters were correlated with the total ASPECTS by the Mann-Whitney non-parametric test. Fisher’s exact test was also used in order to determine the relationship between stenosis and atherosclerotic plaque. Values of *p* ≤ 0.05 were considered statistically significant.

## RESULTS

We evaluated a total of 28 patients with acute/hyperacute stroke. Of those 28 patients, four (14.3%) were under 55 years of age, three (10.7%) were 55-64 years of age, eight (28,6%) were between 65 and 74 years of age, and 13 (46.4%) were ≥ 75 years of age. The mean age was 70 ± 18 years.

Of the 28 patients evaluated, seven (25.0%) had no stenosis, 16 (57.1%) had < 50% stenosis, and five (17.9%) had ≥ 50% (relevant) stenosis. Seven patients (25.0%) had calcified plaques, four (14.3%) had mixed, predominantly calcified plaques, two (7.1%) had mixed, predominantly fatty-fibrous plaques, five (17.9%) had fatty-fibrous plaques, and 10 (35.7%) had no plaques. Among the 18 patients with plaque, it was categorized as stable in six (33.3%) and as vulnerable in 12 (66.6%) Of the 16 patients who had plaques without relevant stenosis, 10 (62.5%) had vulnerable plaques.

Sixteen (57.1%) of the 28 patients had an ASPECTS of 10. Of the 12 remaining patients, only one (8.3%) had an ASPECTS < 5, whereas five (41.7%) had an ASPECTS of 5-7 and six (50.0%) had an ASPECTS ≥ 7.

Of the 28 patients evaluated, six (21.4%) had TACI, 16 (57.1%) had PACI, five (17.9%) had lacunar infarct, and one (3.6%) had infarct in the centrum semiovale. There were no cases of border-zone infarct.

The etiology of cerebral infarct was defined as large-vessel atherosclerosis, cerebral small-vessel disease, cardioembolic stroke, and undetermined cause in 32.1%, 7.1%, 7.1%, and 53.6% of the cases, respectively. No cases fell within the other determined etiology category.

In our sample, four (14.3%) of the patients underwent thrombolytic treatment, seven (25.0%) underwent mechanical thrombectomy, and 17 (60.7%) underwent conservative treatment only.

When we correlated the dichotomized ASPECTS with the other parameters evaluated (Table 1), we found that the dichotomized ASPECTS showed a statistically significant correlation with relevant stenosis and with total infarct. The risks of having relevant stenosis and total infarct were found to be greater among the patients with an ASPECTS ≤ 7 than among those with an ASPECTS > 7 (*p* = 0.022 and *p* < 0.001, respectively).

The correlation between vulnerable plaque and the dichotomized ASPECTS was not statistically significant. However, it is noteworthy that nine patients had no detectable plaque and were therefore excluded from that analysis. Vulnerable plaques were present in 12 (54.5%) of the 22 patients with an ASPECTS > 7 and in none of the patients with an ASPECTS ≤ 7. The dichotomized ASPECTS did not correlate significantly with atherosclerosis as cause or with age.

**Table 1:** Relationship of the dichotomized ASPECTS with the parameters evaluated.

Parameter	ASPECTS ≤ 7 (n = 6)	ASPECTS > 7 (n = 22)	Total (n = 28)	*P*-value*
Relevant (≥ 50%) stenosis, n (%)				
Yes	3 (50.0)	1 (4.5)	4 (14.3)	0.022
No	3 (50.0)	21 (95.5)	24 (85.7)	
Vulnerable plaque^†^, n (%)				
Yes	0 (0)	12 (66.7)	12 (57.1)	0.063
No	3 (100.0)	6 (33.3)	9 (42.9)	
Infarct type, n (%)				
Partial	0 (0)	22 (100.0)	22 (78.6)	< 0.001
Total	6 (100.0)	0 (0)	6 (21.4)	
Specific treatment, n (%)				
Yes	5 (83.3)	6 (27.3)	11 (39.3)	0.022
No	1 (16.7)	16 (72.7)	17 (60.7)	
Atherosclerosis as cause, n (%)				
Yes	2 (33.3)	7 (31.8)	9 (32.1)	0.999
No	4 (66.7)	15 (68.2)	19 (67.9)	
Age, n (%)				
< 65 years	3 (50.0)	4 (18.2)	7 (25.0)	0.144
≥ 65 years	3 (50.0)	18 (81.8)	21 (75.0)	

* Fisher’s exact test;

† Seven cases were not evaluated (no plaque).

**Table 2:** Relationship of the total ASPECTS with the parameters evaluated.

Parameter	n	Total ASPECTS					P-value*
		Mean	Standard deviation	Minimum	Median	Maximum	
Relevant (≥ 50%) stenosis, n (%)						
Yes	4	6.5	1.732	5	6	9	0.008
No	24	9.1	1.909	2	10	10	
Vulnerable plaque^†^, n (%)						
Yes	12	9.7	0.492	9	10	10	0.270
No	9	8.3	2.121	5	10	10	
Infarct type, n (%)						
Partial	22	9.7	0.568	8	10	10	< 0.001
Total	6	5.2	1.602	2	6	6	
Specific treatment, n (%)						
Yes	11	7.8	2.04	5	8	10	0.036
No	17	9.3	1.929	2	10	10	
Atherosclerosis as cause, n (%)						
Yes	9	8.7	1.871	5	9	10	0.529
No	19	8.7	2.207	2	10	10	
Age, n (%)						
< 65 years	7	8.3	2.138	6	10	10	0.745
≥ 65 years	21	8.9	2.081	2	10	10	

* Fisher’s exact test;

† Seven cases were not evaluated (no plaque).

The total ASPECTS showed a statistically significant correlation with relevant stenosis and with total infarct (Table 2). Therefore, the total ASPECTS was lower among the patients with stenosis than among those without (*p* = 0.008), as well as being lower among patients with total infarct than among those with partial infarct (*p* < 0.001).

The total ASPECTS did not correlate significantly with vulnerable plaque, atherosclerosis as cause, or age. In addition, atherosclerosis as cause did not correlate significantly with relevant stenosis or with vulnerable plaque.

**Figure 1:**
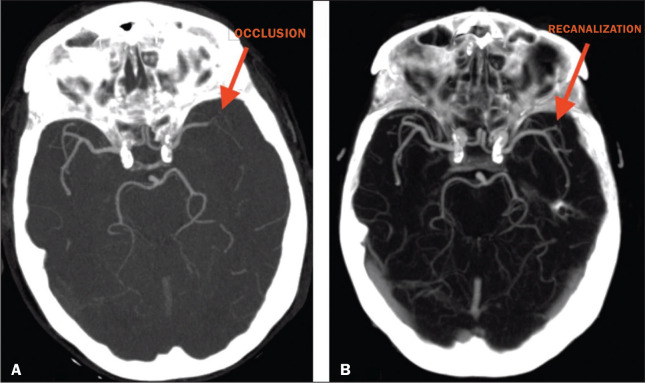
Patient with occlusion in the M2 branches of the left MCA bifurcation (A) and complete recanalization after thrombectomy (B).

**Figure 2:**
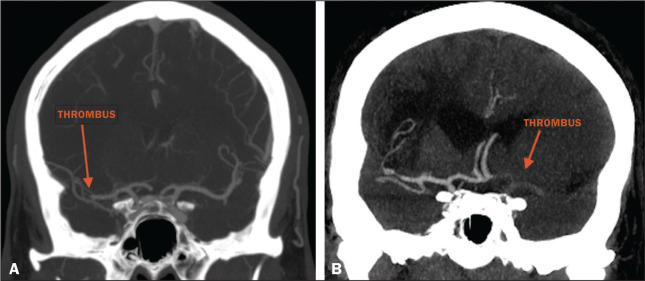
Patient with occlusion in the M2 segment of the right MCA (A); and patient with occlusion in the left ICA bifurcation (B).

In our study sample, an initial unenhanced CT scan showed no visible changes in 15 (53.6%) of the 28 patients, whereas an initial CTA showed changes in eight (53.3%) of those 15 patients, including one who was treated with mechanical thrombectomy. In addition, CTA identified arterial occlusions in 11 (39.3%) of the cases (Figures 1 and 2). In seven of those patients, mechanical thrombectomy was indicated. Three patients had distal occlusions in the branches of the MCA and in the ACA. For those patients, hemodynamic treatment was not indicated.

## DISCUSSION

In cases of stroke, the findings on an unenhanced CT scan acquired early may be normal, ischemic changes being visible only on a subsequent (follow-up) scan^**(2)**^. Our study underscores the importance of including CTA in the stroke protocol, because it provides information complementary to that obtained with unenhanced CT, which can fail to identify early changes. In a study conducted by von Kummer et al.^**(22)**^, unenhanced CT detected no changes in one third of the cases in whom a diagnosis of stroke was subsequently confirmed. However, the authors found that the failure to detect such changes correlated with less severity of ischemia, which leads to late ischemic tissue damage.

The use of CTA adds information that is relevant for identifying occlusion, as well as for evaluating its location and extent, and informs decisions regarding the best therapeutic approach for each patient. Our study confirms the importance of including CTA in the initial protocol for patients with stroke, showing that it can facilitate the indication of mechanical thrombectomy in stroke victims.

It remains unclear whether hemodynamic treatment (mechanical thrombectomy) is beneficial in stroke victims with distal occlusions in certain arterial segments^**(12)**^. The 2018 American Heart Association guidelines on the management of acute stroke classify stent thrombectomy in cases of occlusion in the M2 and M3 segments of the MCA as class IIb evidence (of uncertain benefit), although the therapeutic approach in cases of distal occlusion in the M2 segment is undergoing changes^**(6,12)**^. According to Mokin et al.^**(6)**^ and Powers et al.^**(12)**^, there have been five studies involving patients with occlusion of the M2 segment who underwent thrombectomy—MR CLEAN; ESCAPE; REVASCAT; SWIFT PRIME; and EXTEND-IA—all of which reported favorable results. Due to the lack of studies showing the benefit of thrombectomy for more distal MCA occlusions (M3 segments) or ACA occlusions, it is not yet recommended for use in those locations^**(6)**^.

To determine the best therapeutic approach after stroke, it is important to take into account all of the signs indicative of pathology in the carotid artery. Classically, the parameter of choice was the degree of extracranial stenosis as an indirect indicator of the atherosclerotic process. However, studies have shown that some patients with atherosclerotic disease without relevant stenosis may still be at high risk depending on the plaque morphology, which underscores the importance of direct evaluation of plaque structure and composition in preventing the development of future ischemic events, as well as in guiding treatment^**(22-24)**^.

In the present study, 57.1% of the patients had atheromatous plaques without relevant stenosis, of whom 62.5% had vulnerable plaques. Therefore, if the decision to treat were based only on the parameter of indirect stenosis, 36% of our patients would not receive adequate treatment. The same was observed in the study of Pacheco et al.^**(18)**^, in which 34% of the patients had stenosis that was not considered relevant and 59% of those patients had vulnerable plaques. Therefore, 20% of their patients would not have received adequate treatment if only the criterion of indirect stenosis were considered.

In our study sample, the ASPECTS was lower among the patients with relevant stenosis, who were also more likely to have a total infarct. Yoo et al.^**(10)**^ observed that the incidence of ICA terminus occlusion was higher among patients with lower ASPECTS, further showing that the time from stroke onset to CT was longer for such patients than for those with higher ASPECTS and that delayed treatment has a deleterious effect mediated by expansion of the infarct.

We found no statistically significant correlation between the presence of vulnerable plaque and the dichotomized ASPECTS. However, it should be borne in mind that most of the patients with an ASPECTS ≥ 7 had suffered a stroke of undetermined etiology, without relevant atherosclerosis, which precluded the detection of such a correlation.

In the present study, the most common diagnosis was stroke of undetermined etiology, followed by stroke attributed to large-vessel atherosclerosis. In the patient samples evaluated by Pacheco et al.^**(18)**^ and Silva et al.^**(20)**^, atherosclerosis was the main etiology, identified in 50% and 41% of the cases, respectively. However, other authors have reported large proportions of patients diagnosed with stroke of undetermined etiology, thus highlighting one of the limitations of the TOAST classification system, which includes many heterogeneous conditions in this category, resulting in an overestimation of its incidence^**(25-27)**^.

The mean age of the patients in our sample was 70 years, which is comparable to that reported in similar studies^**(2,13,20)**^. Although cerebral ischemia has been reported to be uncommon in younger stroke patients, recent studies have shown that its incidence has increased and that it now accounts for 5-20% of all strokes among such patients^**(27-30)**^. Yamamoto^**(25)**^ reported that, in clinical practice, it is not uncommon to encounter young stroke victims in whom without the etiology cannot be identified even after extensive clinical evaluation. In a review article authored by Correia et al.^**(28)**^, the cause of stroke was unidentified, even after extensive clinical evaluation, in 30% of the cases. In our study sample, 14.3% of the patients were under the age of 55 years old and it was not possible to accurately identify the ischemic etiology in those patients. One possible explanation is that the etiopathogenesis is related to transient, completely reversible phenomena, and an earlier, even more prolonged investigation would therefore be needed in order to identify those phenomena^**(25,28)**^.

Our study has some limitations. First, our patient sample was small and was limited to only one institution. In addition, our evaluation was restricted to infarcts in the anterior circulation. Furthermore, our investigation was inconclusive because some patients died or were lost to outpatient follow-up before the cause of the infarct could be determined. Nevertheless, we were able to identify a number of interesting relationships among stroke, the ASPECTS, and atherosclerosis. Knowledge of those relationships could facilitate the evaluation of patients with acute stroke.

## CONCLUSION

Our data reveal that CTA of the intracranial and cervical vessels in acute/hyperacute stroke enables the identification of the thrombus, allowing mechanical thrombectomy to be indicated in some patients, and provides better evaluation of plaques. These data show that it is possible to use CTA to evaluate atheromatous plaques early and that their characteristics can correlate with the cause of the ischemic event, the choice of treatments being guided by those characteristics. Therefore, we underscore the importance of adding CTA to the stroke protocol, in order to inform decisions regarding the most appropriate early treatment and treatment for secondary prevention.
